# Influence of Hypertension Management on Survival in Patients With Metastatic Breast Cancer

**DOI:** 10.1002/cam4.71642

**Published:** 2026-02-26

**Authors:** Reina Haque, Amrita Mukherjee, Lie Hong Chen, Tiffany A. Hogan, Moira Brady‐Rogers, Zheng Gu, Ariel Silverman, Ana Barac, Lauren P. Wallner

**Affiliations:** ^1^ Department of Research & Evaluation Kaiser Permanente Southern California Pasadena California USA; ^2^ Department of Health Systems Science Kaiser Permanente Bernard J. Tyson School of Medicine Pasadena California USA; ^3^ Department of Hematology/Oncology Kaiser Permanente Los Angeles Medical Center Los Angeles California USA; ^4^ Alive & Well Women South Pasadena California USA; ^5^ Inova Schar Heart and Vascular Falls Church Virginia USA; ^6^ Georgetown University Washington District of Columbia USA; ^7^ Department of Internal Medicine and Epidemiology University of Michigan Ann Arbor Michigan USA

**Keywords:** antihypertensives, comorbidities, disparities, hypertension, metastatic breast cancer, mortality, survival

## Abstract

**Background:**

Although nearly half of women with metastatic breast cancer (mBC) have hypertension, it is unclear whether hypertension management improves survival. We examined the influence of pharmacologic hypertension management on all‐cause and breast cancer‐specific mortality in patients with mBC.

**Methods:**

We conducted a longitudinal cohort study of 1332 female patients with de novo mBC diagnosed 2008–2020, followed through 2021. We calculated person‐year (PY) rates of all‐cause and breast cancer mortality by use of antihypertensives (monotherapy or polytherapy [one vs. multiple drug classes]). Multivariable Cox regression was used to estimate the association between antihypertensives and mortality.

**Results:**

Overall, 48.4% of patients with mBC had hypertension, which was greatest in Black women (64.6%). During follow‐up, 52.9% were treated with antihypertensive medications (20.3% monotherapy; 32.5% polytherapy). All‐cause mortality rates were lower in the polytherapy (21.4/100 PY) versus monotherapy (28.5/100 PY) group. All‐cause mortality risk was 38% lower (adjusted HR = 0.62; 95% CI: 0.47–0.82) in the polytherapy group vs. monotherapy. This protection was significantly greater in Hispanic patients (HR = 0.40; 95% CI: 0.20–0.84) and suggested in Black patients (HR = 0.48; 95% CI: 0.22–1.05). Similarly, breast cancer mortality was lower in those treated with polytherapy versus monotherapy, particularly those with good medication adherence (HR = 0.43; 95% CI: 0.24–0.77).

**Conclusion:**

In patients with mBC, all‐cause mortality risk was lower among those treated with antihypertensive polytherapy versus monotherapy, with the greatest risk attenuation seen among Hispanic women. Additional prospective studies are needed to examine comorbidity management strategies that may help patients with mBC extend life, particularly including women of color.

## Graphical abstract

In a cohort of 1332 patients with metastatic breast cancer, 52.9% were treated with antihypertensive medications (20.3% monotherapy; 32.5% polytherapy). All‐cause mortality risk was 38% lower (adjusted HR; 95% CI: 0.62; 0.47–0.82) in the antihypertensive polytherapy group vs. monotherapy, and this protection was significantly greater in Hispanic patients (HR = 0.40; 95% CI: 0.20–0.84) and suggested in Black patients (HR = 0.48; 95% CI: 0.22–1.05).

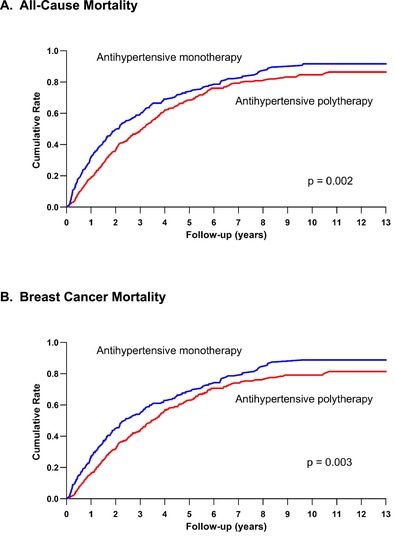

## 1 Introduction

Nearly 6%–10% of women with breast cancer are diagnosed with de novo stage IV disease in the U.S., while 20%–30% of women with early‐stage breast cancer later develop metastatic breast cancer (mBC) [1]. Despite medical advances and improvements in mortality rates, racial disparities persist in breast cancer outcomes. In fact, among non‐Hispanic Black (NHB) women, mBC represents 9% of diagnoses in comparison to 5%–6% in other racial/ethnic groups [2, 3]. While breast cancer mortality rates have been declining over time, the decline is not equivalent across racial and ethnic groups [4]. Reasons for this are not clear, and may be influenced by tumor biology [5], comorbidities, social determinants of health, and implicit bias in healthcare settings. National cancer registry data from 2009 to 2015 shows that the 5‐year mBC‐specific survival rate is just 21% among Black women compared to 28% for White women [2, 3]. Even less is known about women from other racial/ethnic backgrounds.

At time of breast cancer diagnosis, women often present with one or more comorbidities, such as hypertension, obesity, or diabetes [6, 7, 8]. Some of the observed racial/ethnic survival disparities may contribute to differential burdens of comorbidities after breast cancer diagnosis. Hypertension is the most common comorbidity (~60% prevalence) and disproportionately affects Black women [9]. Prior studies suggest hypertension is associated with treatment delays, more hospitalizations, and higher mortality in patients with early‐stage breast cancer. Braithwaite et al. found that Charlson Comorbidity Index (CCI) and hypertension were independently associated with lower overall and non‐breast cancer survival in women with early‐stage breast cancer [10], and that hypertension had prognostic significance for the survival disparity between Black and White patients [11].

To date, no studies have examined the influence of hypertension on outcomes after diagnosis in patients with mBC. The influence of hypertension and its pharmacological management on mortality, and their contribution to survival disparities in patients with mBC, remains unknown. To address this gap, we explored the influence of hypertension on all‐cause mortality in a diverse cohort of women with mBC, and for the first time, examined if pharmacologic management of hypertension can reduce mortality risk.

## 2 Materials & Methods

### 2.1 Study Design, Setting, and Subjects

This longitudinal cohort included patients diagnosed with de novo mBC between 2008 and 2020 at Kaiser Permanente Southern California (KPSC), a not‐for‐profit integrated healthcare delivery system comprised of 16 community hospitals and over 200 medical offices geographically spread across Southern California, serving over 4.9 million members. KPSC patients receive virtually all their medical care, including pharmacy prescriptions, within this healthcare system. Data on cancer‐related variables, sociodemographic, and clinical variables were extracted from KPSC's cancer registry, pharmacy dispensing, and electronic health records (Figure S1). Rare medical procedures and hospitalizations outside of the system were captured from claims databases. Women diagnosed with de novo mBC were identified from the KPSC's NCI's‐Surveillance Endpoints and End Results (SEER)‐affiliated cancer registry. The study was reviewed by the KPSC Internal Review Board, which waived the right to obtain written or verbal consent from patients for the de‐identified analytic dataset. Females aged ≥ 18 years who were newly diagnosed with stage IV breast cancer (American Joint Commission on Cancer TNM) in 2008–2020 were included in this analysis (*N* = 1332). No other exclusionary factors were applied to enhance generalizability.

### 2.2 All‐Cause Mortality Outcome

Date and causes of death were identified from inpatient databases and National Death Index databases (using social security number linkages) for deaths that occurred outside of the KPSC system to mitigate loss to follow‐up. The main study outcome was all‐cause (overall) mortality. Patients were followed from mBC diagnosis until patients died or reached the study's end (December 31, 2021), whichever occurred first.

### 2.3 Antihypertensive Pharmacy Data

Our primary exposure variable was use of antihypertensive drugs after mBC diagnosis identified from the pharmacy dispensing database, and patients had to have at least two dispensings to be considered exposed. Major classes of antihypertensive drugs were: angiotensin‐converting enzyme inhibitors (ACEIs), Angiotensin Receptor Blockers (ARBs), beta blockers (BBs), calcium channel blockers (CCBs), diuretics, vasodilators, other (e.g., alpha blockers), and combinations. Subjects were classified into monotherapy (used one class of antihypertensives) and polytherapy (used multiple classes including single‐pill combinations) to minimize confounding by indication. Polytherapy is typically prescribed when hypertension becomes severe or when a single drug class becomes ineffective. Common polytherapy regimens include combining drugs from different classes like ACEIs or Angiotensin Receptor Blockers (ARBs) with CCBs or thiazide diuretics [12]. Therapy was handled as time‐dependent, that is, women were categorized as monotherapy users until they filled a prescription from a different class, and this variable was updated every 6 months. Pre‐existing hypertension prevalence was ascertained up to one‐year prior to mBC diagnosis date as described below [13].

To evaluate adherence to antihypertensives, medication possession ratio (MPR) was calculated as the number of days supplied (excluding last refill) divided by the number of days between first and last dispense dates over the study follow‐up period. The MPR ≥ 80% is an established level that reflects there are very few days without drugs on hand, and hence, continuous medication usage [14].

### 2.4 Covariates

We extracted the most common comorbidities (hypertension, diabetes, dyslipidemia, obesity) up to 1 year before mBC diagnosis from electronic health records, using an algorithm of ICD9CM and ICD10CM codes successfully used in our prior studies [13]. Additional comorbidities were also examined using the Elixhauser Comorbidity Index (ECI) [15]. Blood pressure measurements from encounters during follow‐up were extracted. Medications for other cardiometabolic conditions, including antidiabetics and antilipemics (statins), were also extracted. We calculated an annualized number of outpatient visits to adjust for overall healthcare utilization to address potential confounding from the greater frequency of outpatient visits being correlated with more monitoring and comorbidity management.

We ascertained race/ethnicity from the cancer registry: (Asian/Pacific Islander [API], non‐Hispanic African American/Black [Black], non‐Hispanic White [NHW], Hispanic, and Other/Mixed/Native American patients), and palliative cancer treatments (hormonal, chemotherapy, other systemic therapy and radiation). Other covariates included: age and year of mBC diagnosis, neighborhood deprivation index and geocoded median household income (as measures of socioeconomic status [SES]) based on the U.S. 2010 Census data [16], insurance payor, smoking history, physical activity, and body mass index (BMI) closest to the initial mBC diagnosis.

Information on first‐course cancer therapy in the first 6 months after diagnosis was extracted from the cancer registry. We also captured intrinsic subtype based on the combination of estrogen (ER), progesterone (PR), and HER‐2 markers (triple‐negative, luminal A, luminal B, HER‐2 enriched), and tumor size.

### 2.5 Statistical Analysis

Distributions of sociodemographic and clinical factors by race/ethnicity were first examined with frequencies and proportions for categorical variables using chi‐square test or Fisher exact test and medians for continuous variables (time to treatments) using Kruskal–Wallis test. Similarly, use of antihypertensive, antidiabetic, and statin use (ever/never categories) was examined by race/ethnicity. Follow‐up time commenced on the mBC diagnosis date and ended on the date of death or study's end date (December 31, 2021), whichever occurred first. Because patients had varying follow‐up lengths, we calculated person‐year (PY) mortality rates by race/ethnicity and by antihypertensive medication use (monotherapy or polytherapy).

Multivariable Cox proportional hazard regression was used to estimate the association (hazard ratio [HR], 95% confidence interval [CI]) between overall mortality and antihypertensive use (monotherapy vs. polytherapy). Antihypertensive use status (monotherapy or polytherapy) was handled as a binary time‐dependent variable (i.e., 0 up to start date and 1 after start date of each therapy) as were statins and antidiabetic medications use. All medication variables were updated every 6 months. We used the Least Absolute Shrinkage and Selection Operator method (LASSO) for initial variable selection [17]. Final variables were selected based on the clinical importance, and on a combination of information criteria like Akaike Information Criterion (AIC) and Bayesian Information Criterion (BIC) to penalize model complexity and choose the best model.

We also ran Cox proportional hazard models incorporating inverse probability of treatment weighting (IPTW) based on the propensity scores (PS) to address potential selection bias (PS Model). PS were constructed using a multivariable logistic regression model using the baseline covariates mentioned above and represented the probability of receiving polytherapy or monotherapy of antihypertensive medications.

Additionally, we examined mortality risk by medication adherence computed MPR in separate Cox models. The proportional hazard assumption was tested via graphic plots and residual analysis. No violations were found. The percentage of missingness was low (<5%) for most of the covariates, except for breast cancer subtypes. Therefore, missing values were handled as an additional category in the models.

To explore if blood pressure control differed between patients with antihypertensive polytherapy and monotherapy, we examined the percentage of patients with baseline hypertension (*N* = 482) who achieved systolic blood pressure <140 mmHg over any continuous 6‐month period during follow‐up in a sensitivity analysis. As research demonstrates that systolic blood pressure is predictive of future cardiovascular events and overall deaths [18], this cut‐off (<140 mmHg) was based on National Committee for Quality Assurance (NCQA) specifications [19] and prior literature [20]. All analyses were performed with SAS 9.4 (SAS Institute Inc).

## 3 Results

This longitudinal cohort included *N* = 1332 female patients diagnosed with de novo mBC between 2008 and 2020 and followed through December 2021. Median age was 64 years at diagnosis (interquartile range [IQR]: 53–75 years). Median years of survival was 1.9 (IQR: 0.8–4.0 years), and the maximum study follow‐up was 13.9 years. The cohort consisted of 46.2% people of color. Overall, 48.4% of patients had hypertension at mBC diagnosis, with Black patients having the highest (64.6%); followed by non‐Hispanic White (NHW 46.3%); Hispanic (46.1%); and API patients (42.8%) (Table 1). Diabetes prevalence was 19.9% overall. Black patients had the highest diabetes prevalence (27.5%). However, Black and NHW patients had a similar prevalence of dyslipidemia (about 44.0% in both). Based on geocoded data at the block level, Black and Hispanic patients were more likely to live in areas with the lowest median household income. API subjects tended to be diagnosed at earlier ages and have fewer comorbidities such as hypertension, diabetes, and obesity (*p* < 0.001 for all variables). The overall prevalence of triple‐negative tumors was 14.3% with Black patients having the highest (18.0%). We found little difference in types of palliative treatments across race and ethnicity.

**TABLE 1:** Demographic and clinical characteristics of patients with de novo stage IV metastatic breast cancer diagnosed between 2008 and 2020.

	Asian/Pacific Islander (*N* = 138)	Black Non‐Hispanic (*N* = 189)	Hispanic (*N* = 258)	White Non‐Hispanic (*N* = 717)	Other/mixed/unknown (*N* = 30)	Total (*N* = 1332)	*p* *
Age at diagnosis (years)							
< 40	17 (12.3%)	6 (3.2%)	13 (5.0%)	33 (4.6%)	0 (0%)	69 (5.2%)	< 0.001
40–49	25 (18.1%)	28 (14.8%)	55 (21.3%)	71 (9.9%)	3 (10.0%)	182 (13.7%)	
50–59	29 (21.0%)	35 (18.5%)	59 (22.9%)	125 (17.4%)	15 (50.0%)	263 (19.7%)	
60–69	32 (23.2%)	43 (22.8%)	54 (20.9%)	168 (23.4%)	3 (10.0%)	300 (22.5%)	
70–79	25 (18.1%)	45 (23.8%)	44 (17.1%)	186 (25.9%)	8 (26.7%)	308 (23.1%)	
80+	10 (7.3%)	32 (16.9%)	33 (12.8%)	134 (18.7%)	1 (3.3%)	210 (15.8%)	
High school graduate and above							
0%–50% (HS grad)	0 (0%)	19 (10.1%)	24 (9.3%)	6 (0.8%)	0 (0%)	49 (3.7%)	< 0.001
51%–75% (Some college)	37 (26.8%)	66 (34.9%)	86 (33.3%)	111 (15.5%)	9 (30.0%)	309 (23.2%)	
76%–100% (College)	101 (73.2%)	104 (55.0%)	148 (57.4%)	600 (83.7%)	21 (70.0%)	974 (73.1%)	
Median household income							
Lowest 20% ($0–$44,539)	20 (14.5%)	80 (42.3%)	56 (21.7%)	95 (13.3%)	7 (23.3%)	258 (19.4%)	< 0.001
20%–40% (>$44,53–$58,043)	29 (21.0%)	34 (17.9%)	50 (19.4%)	117 (16.3%)	9 (30.0%)	239 (17.9%)	
40%–60% (>$58,043–$71,928)	24 (17.4%)	26 (13.8%)	53 (20.5%)	136 (18.9%)	2 (6.7%)	241 (18.1%)	
60%–80% (>$71,928–$91,190)	32 (23.2%)	31 (16.4%)	55 (21.3%)	183 (25.5%)	4 (13.3%)	305 (22.9%)	
Top 20% (>$91,190)	33 (23.9%)	18 (9.5%)	44 (17.1%)	186 (25.9%)	8 (26.7%)	289 (21.7%)	
Neighborhood Deprivation Index							
Median (Q1, Q3)	−0.1 (−0.5, 0.5)	0.6 (−0.1, 1.6)	0.2 (−0.3, 1.0)	−0.3 (−0.7, 0.3)	0.4 (−0.3, 0.9)	−0.1 (−0.6, 0.6)	< 0.001
Year of initial BC dx							
2008	9 (6.5%)	13 (6.9%)	12 (4.6%)	45 (6.3%)	1 (3.3%)	80 (6.0%)	0.11
2009–2010	18 (13.0%)	35 (18.5%)	24 (9.3%)	95 (13.3%)	5 (16.7%)	177 (13.3%)	
2011–2012	11 (7.9%)	29 (15.3%)	30 (11.6%)	97 (13.5%)	2 (6.7%)	169 (12.7%)	
2013–2014	18 (13.0%)	27 (14.3%)	37 (14.3%)	86 (11.9%)	3 (10.0%)	171 (12.8%)	
2015–2016	15 (10.9%)	19 (10.1%)	44 (17.1%)	124 (17.3%)	5 (16.7%)	207 (15.5%)	
2017–2018	36 (26.1%)	29 (15.3%)	55 (21.3%)	148 (20.6%)	5 (16.67%)	273 (20.5%)	
2019–2020	31 (22.5%)	37 (19.6%)	56 (21.7%)	122 (17.0%)	9 (30.0%)	255 (19.1%)	
Elixhauser Comorbidity Index							
0	41 (29.7%)	29 (15.3%)	43 (16.7%)	148 (20.6%)	9 (30.0%)	270 (20.3%)	< 0.001
1	21 (15.2%)	29 (15.3%)	66 (25.6%)	130 (18.1%)	6 (20.0%)	252 (18.9%)	
2	29 (21.0%)	27 (14.3%)	37 (14.3%)	125 (17.4%)	6 (20.0%)	224 (16.8%)	
3	16 (11.6%)	24 (12.7%)	41 (15.9%)	86 (11.9%)	4 (13.3%)	171 (12.8%)	
4	14 (10.1%)	19 (10.1%)	24 (9.3%)	69 (9.6%)	0 (0%)	126 (9.5%)	
5+	16 (11.6%)	59 (31.2%)	44 (17.1%)	154 (21.5%)	2 (6.7%)	275 (20.7%)	
Missing	1 (0.7%)	2 (1.1%)	3 (1.2%)	5 (0.7%)	3 (10.0%)	14 (1.1%)	
Diabetes							
No	111 (80.4%)	135 (71.4%)	189 (73.3%)	596 (83.1%)	22 (73.3%)	1053 (79.1%)	< 0.001
Yes	26 (18.8%)	52 (27.5%)	66 (25.6%)	116 (16.2%)	5 (16.7%)	265 (19.9%)	
Missing	1 (0.7%)	2 (1.1%)	3 (1.2%)	5 (0.7%)	3 (10.0%)	14 (1.1%)	
Hypertension							
No	78 (56.5%)	65 (34.4%)	136 (52.7%)	380 (53.0%)	14 (46.7%)	673 (50.5%)	< 0.001
Yes	59 (42.8%)	122 (64.6%)	119 (46.1%)	332 (46.3%)	13 (43.3%)	645 (48.4%)	
Missing	1 (0.7%)	2 (1.1%)	3 (1.2%)	5 (0.7%)	3 (10.0%)	14 (1.1%)	
Dyslipidemia							
No	87 (63.0%)	106 (56.1%)	153 (59.3%)	397 (55.4%)	22 (73.3%)	765 (57.4%)	0.16
Yes	51 (36.9%)	83 (43.9%)	105 (40.7%)	320 (44.6%)	8 (26.7%)	567 (42.6%)	
Depression							
No	127 (92.0%)	157 (83.1%)	218 (84.5%)	574 (80.1%)	26 (86.7%)	1102 (82.7%)	< 0.001
Yes	10 (7.3%)	30 (15.9%)	37 (14.3%)	138 (19.3%)	1 (3.3%)	216 (16.2%)	
Missing	1 (0.7%)	2 (1.1%)	3 (1.2%)	5 (0.7%)	3 (10.0%)	14 (1.1%)	
Chronic Kidney Disease							
No	122 (88.4%)	157 (83.1%)	211 (81.8%)	595 (82.9%)	26 (86.7%)	1111 (83.4%)	0.51
Yes	16 (11.6%)	32 (16.9%)	47 (18.2%)	122 (17.1%)	4 (13.3%)	221 (16.6%)	
BMI (kg/m^2^)							
<18.5 (Underweight)	4 (2.9%)	14 (7.4%)	2 (0.8%)	23 (3.2%)	2 (6.7%)	45 (3.4%)	< 0.001
18.5 to 24.9 (Healthy)	67 (48.6%)	42 (22.2%)	69 (26.7%)	226 (31.5%)	11 (36.7%)	415 (31.2%)	
25 to 29.9 (Overweight)	42 (30.4%)	43 (22.8%)	81 (31.4%)	206 (28.7%)	7 (23.3%)	379 (28.5%)	
30 or more (Obese)	25 (18.1%)	90 (47.6%)	106 (41.1%)	259 (36.1%)	9 (30.0%)	489 (36.7%)	
Missing	0 (0%)	0 (0%)	0 (0%)	3 (0.4%)	1 (3.3%)	4 (0.3%)	
Physical activity							
Yes (>1×/week)	62 (44.9%)	62 (32.8%)	93 (36.1%)	235 (32.8%)	12 (40.0%)	464 (34.8%)	0.25
No	71 (51.5%)	115 (60.85%)	155 (60.1%)	449 (62.2%)	16 (53.3%)	806 (60.5%)	
Missing	5 (3.6%)	12 (6.4%)	10 (3.9%)	33 (4.6%)	2 (6.7%)	62 (4.7%)	
Insurance payor							
Medicaid	1 (0.7%)	2 (1.1%)	12 (4.7%)	11 (1.5%)	0 (0%)	26 (1.9%)	< 0.001
Medicare	39 (28.3%)	75 (39.7%)	82 (31.8%)	371 (51.7%)	10 (33.3%)	577 (43.3%)	
Medicare and Medicaid	2 (1.5%)	5 (2.7%)	8 (3.1%)	7 (0.9%)	0 (0%)	22 (1.7%)	
Other (commercial, self pay, other)	96 (69.6%)	107 (56.6%)	156 (60.5%)	328 (45.8%)	20 (66.7%)	707 (53.1%)	
Smoking status							
Current smoker	5 (3.6%)	15 (7.9%)	14 (5.4%)	51 (7.1%)	2 (6.7%)	87 (6.5%)	< 0.001
Former smoker	16 (11.6%)	44 (23.3%)	33 (12.8%)	204 (28.5%)	3 (10.0%)	300 (22.5%)	
Never smoker	102 (73.9%)	114 (60.3%)	198 (76.7%)	413 (57.6%)	15 (50.0%)	842 (63.2%)	
Unknown	15 (10.9%)	16 (8.5%)	13 (5.0%)	49 (6.8%)	10 (33.3%)	103 (7.7%)	
Subtypes of breast cancer							
Triple‐negative	16 (11.6%)	34 (18.0%)	35 (13.6%)	104 (14.5%)	2 (6.7%)	191 (14.3%)	0.01
Luminal A	74 (53.6%)	93 (49.2%)	144 (55.8%)	391 (54.5%)	14 (46.7%)	716 (53.8%)	
Luminal B	16 (11.6%)	14 (7.4%)	33 (12.8%)	84 (11.7%)	3 (10.0%)	150 (11.3%)	
HER2 enriched	19 (13.8%)	20 (10.6%)	30 (11.6%)	52 (7.3%)	7 (23.3%)	128 (9.6%)	
HER2 missing & HR+	5 (3.6%)	8 (4.2%)	5 (1.9%)	31 (4.3%)	3 (10.0%)	52 (3.9%)	
HER2 missing & HR‐	0 (0%)	2 (1.1%)	0 (0%)	5 (0.7%)	1 (3.3%)	8 (0.6%)	
Missing/test(s) not done	8 (5.8%)	18 (9.5%)	11 (4.3%)	50 (6.9%)	0 (0%)	87 (6.5%)	
Palliative cancer treatment							
Surgery	45 (32.6%)	49 (25.9%)	77 (29.8%)	204 (28.5%)	3 (10.0%)	378 (28.4%)	0.13
Radiation	34 (24.6%)	35 (18.5%)	62 (24.0%)	154 (21.5%)	6 (20.0%)	291 (21.9%)	0.61
Hormonal therapy	66 (47.8%)	75 (39.7%)	131 (50.8%)	363 (50.6%)	14 (46.7%)	649 (48.7%)	0.29
Chemotherapy	94 (68.1%)	100 (52.9%)	185 (71.7%)	410 (57.2%)	23 (76.7%)	812 (60.9%)	< 0.001
Immunotherapy	24 (17.4%)	17 (8.9%)	41 (15.9%)	80 (11.2%)	9 (30.0%)	171 (12.8%)	< 0.01
Median time to palliative treatments (days between mBC diagnosis & cancer treatment [Q1, Q3])							
Surgery	57.0 (35.0, 153.0)	51.0 (24.0, 130.0)	42.0 (27.0, 140.0)	42.0 (26.0, 138.0)	202.0 (30.0, 234.0)	43.0 (27.0, 143.0)	0.68
Radiation	102.0 (46.5, 226.0)	94.0 (51.0, 195.0)	97.5 (31.0, 237.5)	84.0 (41.5, 201.5)	48.5 (33.0, 339.0)	87.0 (39.5, 220.5)	0.94
Hormonal	48.0 (21.0, 130.0)	51.0 (29.0, 121.0)	55.0 (27.0, 134.0)	47.0 (23.0, 112.0)	73.0 (32.0, 148.0)	49.0 (24.0, 119.5)	0.36
Chemotherapy	42.0 (27.0, 70.0)	44.0 (28.0, 72.0)	45.0 (27.0, 84.5)	41.0 (24.0, 70.0)	32.0 (20.0, 43.0)	42.0 (25.0, 73.0)	0.07
Immunotherapy	51.0 (24.5, 69.5)	54.0 (31.0, 119.0)	39.0 (20.0, 62.0)	48.0 (30.0, 77.5)	43.0 (28.0, 77.0)	45.0 (27.0, 74.0)	0.39
Annualized outpatient visits							
Median (Q1, Q3)	26.9 (19.6, 37.3)	25.9 (16.1, 37.4)	30.0 (19.4, 41.7)	25.5 (16.6, 38.7)	26.3 (15.2, 36.0)	26.5 (17.4, 39.1)	0.09

*Note:* Luminal A (ER+ or PR+ HER2−); Luminal B (ER+ or PR+ HER2−); HER2 enriched (ER− & PR‐ HER2+); HER2 missing & HR+ (ER+ or PR+); HER2 missing & HR− (ER− & PR−).

* *p*‐values are two‐sided.

During follow‐up, 52.9% (*N* = 704) were treated with antihypertensive medications (20.4% received monotherapy and 32.5% received polytherapy) (Table 2). Use of antihypertensives (both monotherapy and polytherapy) did not vary significantly by race and ethnicity. Use of antilipemics was greater in Black patients (27.0%) and NHW patients (29.9%), while use of antidiabetic drugs was greater in Hispanic patients (15.1%) and API patients (13.1%).

**TABLE 2:** Use of cardioprotective medications during follow‐up in patients with de novo metastatic stage IV breast cancer.

	Asian/Pacific Islander (*N* = 138)	Black non‐Hispanic (*N* = 189)	Hispanic (*N* = 258)	White non‐Hispanic (*N* = 717)	Other/mixed/unknown (*N* = 30)	Total (*N* = 1332)	*p* *
Antihypertensives							
No	72 (52.2%)	79 (41.8%)	123 (47.7%)	336 (46.9%)	18 (60.0%)	628 (47.2%)	0.23
Yes	66 (47.8%)	110 (58.2%)	135 (52.3%)	381 (53.1%)	12 (40.0%)	704 (52.9%)	
Monotherapy	28 (20.3%)	38 (20.11%)	46 (17.8%)	154 (21.5%)	5 (16.7%)	271 (20.3%)	0.57
Polytherapy	38 (27.5%)	72 (38.1%)	89 (34.5%)	227 (31.7%)	7 (23.3%)	433 (32.5%)	
MPR < 80%	46 (69.7%)	84 (76.4%)	108 (80.0%)	301 (79.0%)	8 (66.7%)	547 (77.7%)	0.38
MPR ≥ 80%	20 (30.3%)	26 (23.6%)	27 (20.0%)	80 (21.0%)	4 (33.3%)	157 (22.3%)	
Antilipemics (statins)							
No	108 (78.3%)	138 (73.0%)	198 (76.7%)	503 (70.2%)	26 (86.7%)	973 (73.1%)	0.05
Yes	30 (21.7%)	51 (27.0%)	60 (23.3%)	214 (29.9%)	4 (13.3%)	359 (26.9%)	
Antidiabetics							
No	120 (86.9%)	167 (88.4%)	219 (84.9%)	656 (91.5%)	26 (86.7%)	1188 (89.2%)	0.04
Yes	18 (13.1%)	22 (11.6%)	39 (15.1%)	61 (8.5%)	4 (13.3%)	144 (10.8%)	

* Two‐sided *p*‐value was calculated using Chi‐square test.

**FIGURE 1:**
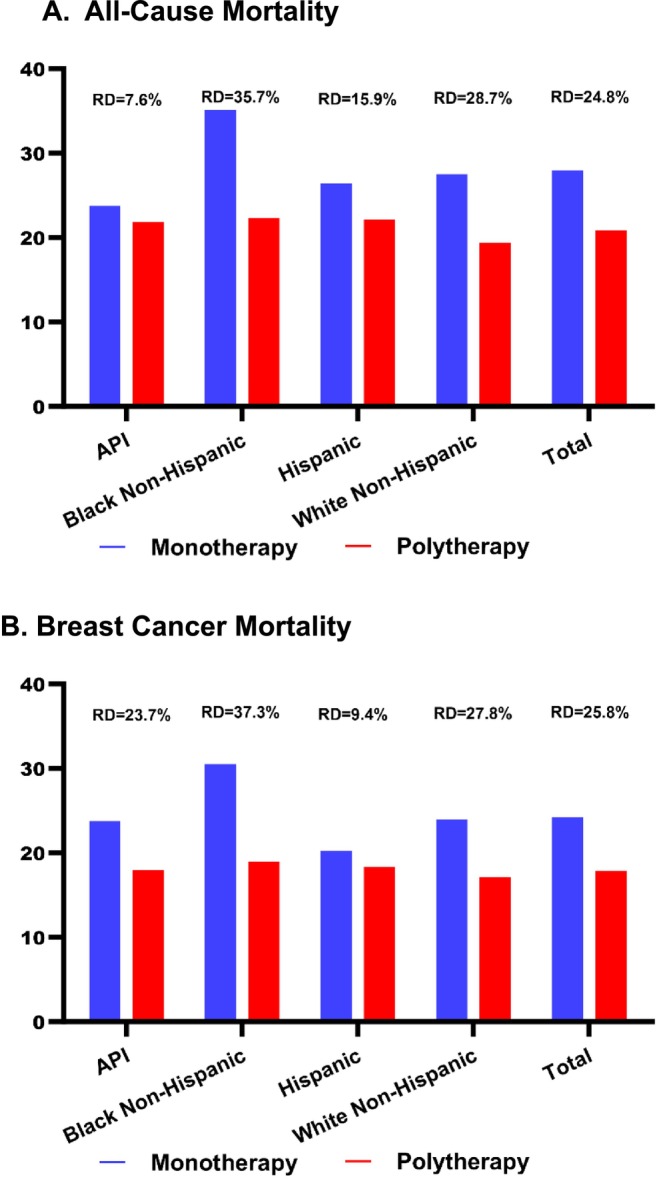
Risk difference percent (RD, %) in (A) all‐cause and (B) breast cancer mortality rates per 100 person‐years by antihypertensive monotherapy and polytherapy status by race/ethnicity in patients with de novo stage IV breast cancer*. *Results for women in the Other/Mixed/Unknown group (*n* = 12) are not shown due to small numbers. RD, Risk difference between monotherapy and polytherapy.

Among those treated with antihypertensives, 73.2% (*N* = 515) patients died due to all‐causes, and the majority was due to breast cancer (*N* = 444). All‐cause mortality rates were lower in those treated with antihypertensive polytherapy (21.4/100 PY) vs. monotherapy (28.5/100 PY) (Table S1). Compared to monotherapy, the percent all‐cause mortality rate reduction (measured by the rate difference) was greater in those treated with polytherapy, particularly in Black patients (35.7% reduction), followed by NHW (28.7%), Hispanic (15.9%), and API patients (7.6%) (*p* < 0.05) (Figure 1A). Breast cancer mortality was also mitigated in the polytherapy group (Figure 1B).

**FIGURE 2:**
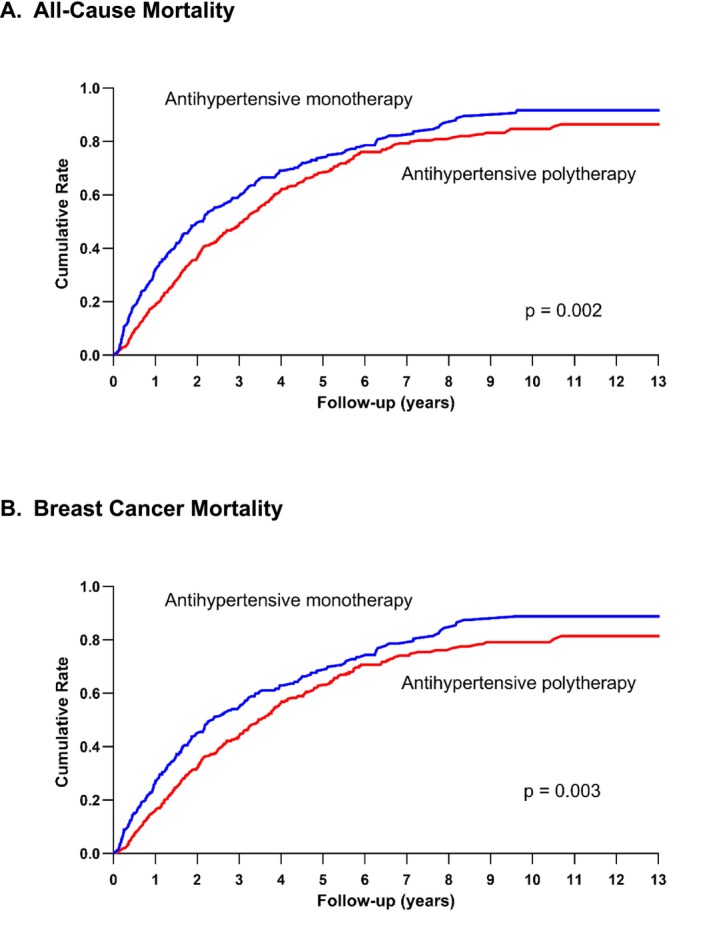
Plot showing cumulative (A) all‐cause and (B) breast cancer mortality risk by antihypertensive monotherapy and polytherapy use.

Table 3 presents the crude and adjusted hazard ratios for the association between antihypertensive polytherapy and monotherapy and mortality risk by race and ethnicity. Antihypertensive polytherapy and monotherapy were handled as time‐varying variables, and the multivariable model adjusted for the aforementioned covariates. All‐cause mortality risk was 38% lower (adjusted HR = 0.62; 95% CI: 0.47–0.82) among those with polytherapy antihypertensive use vs. monotherapy. This protection was even greater in Hispanic patients (adjusted HR = 0.40; 95% CI: 0.20–0.84), and we found a non‐significant decreased risk in Black patients (adjusted HR = 0.48; 95% CI: 0.22–1.05). Hazard ratios for breast cancer mortality were similar to results of all‐cause mortality in the race and ethnic groups, except among API patients in whom we observed wide confidence intervals. Additionally, results from the multivariable model were similar to those from the propensity score methods. Consistent with this, cumulative mortality was also lower in the group exposed to antihypertensive polytherapy during follow‐up as compared with monotherapy (Figure 2). Further, in a sensitivity analysis, we also examined mortality risk by MPR. The protection conferred by better antihypertensive adherence became even stronger in the polytherapy group. Among patients with good adherence to antihypertensives (MPR ≥ 80%), the risk of all‐cause mortality was 58% lower in the polytherapy group (adjusted HR = 0.42; 95% CI: 0.24–0.76) vs. monotherapy group. Similarly, among those with MPR ≥ 80%, the breast cancer mortality risk was 57% lower (adjusted HR = 0.43; 95% CI: 0.24–0.77) in the polytherapy group (Table S2).

**TABLE 3:** Risk of all‐cause and breast cancer mortality in patients with de novo stage IV metastatic breast cancer by race and ethnicity among those treated with antihypertensive medications.

	Total^a^	Asian/Pacific Islander	Black Non‐Hispanic	Hispanic	White Non‐Hispanic
	(*N* = 704)	(*N* = 66)	(*N* = 110)	(*N* = 135)	(*N* = 381)
	HR (95% CI)	HR (95% CI)	HR (95% CI)	HR (95% CI)	HR (95% CI)
All‐cause mortality					
Crude^b^					
Monotherapy	Reference	Reference	Reference	Reference	Reference
Polytherapy	0.55 (0.42–0.72)	0.73 (0.31–1.68)	0.41 (0.22–0.80)	0.55 (0.30–1.03)	0.58 (0.41–0.84)
Adjusted^c^					
Monotherapy	Reference	Reference	Reference	Reference	Reference
Polytherapy	0.62 (0.47–0.82)	1.80 (0.47–6.85)	0.48 (0.22–1.05)	0.40 (0.20–0.84)	0.70 (0.48–1.02)
Adjusted^d^					
Monotherapy	Reference	Reference	Reference	Reference	Reference
Polytherapy	0.58 (0.46–0.75)	1.09 (0.51–2.34)	0.68 (0.42–1.11)	0.51 (0.30–0.87)	0.63 (0.45–0.88)
Breast Cancer mortality					
Crude^b^					
Monotherapy	Reference	Reference	Reference	Reference	Reference
Polytherapy	0.55 (0.42–0.72)	0.59 (0.23–1.49)	0.36 (0.18–0.75)	0.69 (0.35–1.36)	0.59 (0.40–0.86)
Adjusted^c^					
Monotherapy	Reference	Reference	Reference	Reference	Reference
Polytherapy	0.64 (0.47–0.85)	1.13 (0.31–4.09)	0.44 (0.18–1.06)	0.61 (0.28–1.31)	0.71 (0.48–1.06)
Adjusted^d^					
Monotherapy	Reference	Reference	Reference	Reference	Reference
Polytherapy	0.60 (0.46–0.78)	0.96 (0.44–2.08)	0.62 (0.37–1.06)	0.67 (0.37–1.21)	0.62 (0.43–0.89)

^a^ Patients in the Other/Mixed/Unknown group are not shown in this table due to small sample (*n* = 12).

^b^ Crude model based on time‐varying antihypertensive use groups.

^c^ Parsimonious model based on time‐varying antihypertensive groups and adjusted for time‐dependent use of antilipemic and antidiabetic drugs, and baseline age, SES, body mass index, dyslipidemia, Elixhauser comorbidity index, breast cancer subtype, tumor size, Neighborhood Deprivation Index, palliative cancer treatments, insurance payor, and annualized outpatient visits.

^d^ Propensity weighted model: time‐varying antihypertensive groups and adjusted for time‐dependent status of antilipemic and antidiabetic use. Propensity scores were based on multivariable logistic regression using variables selected in the LASSO model.

In another sensitivity analysis based on patients with hypertension at baseline and treated with antihypertensives (*N* = 482) revealed the percentage who achieved <140 mmHg systolic blood pressure over any 6‐month period during follow‐up was 47.5%. Specifically, the percentage who achieved <140 mmHg systolic blood pressure was greater among those treated with polytherapy (75.1%) versus monotherapy (24.9%), corresponding with a 36% lower likelihood of high blood pressure among those with polytherapy (odds ratio [OR] = 0.64; 95% CI: 0.42–0.97) (Table 4). Most of the patients treated with BBs (which may have antitumor effects) were in the antihypertensive polytherapy group (81.2%) compared with the monotherapy group (18.8%) (Table S3).

**TABLE 4:** Percentage of patients with baseline hypertension who achieved <140 mmHg systolic blood pressure over any 6‐month period during follow‐up by antihypertensive monotherapy and polytherapy status.

	Antihypertensive monotherapy^a^ *N* (%)	Antihypertensive polytherapy^a^ *N* (%)	OR (95% CI)
Achieved <140 mmHg systolic pressure			
Yes	57 (24.9%)	172 (75.1%)	0.64 (0.42–0.97)
No	86 (34.0%)	167 (66.0%)	1.00 (Reference)

Abbreviation: OR, overall odds ratio.

^a^ Row percent.

## 4 Discussion

In this diverse cohort of women with de novo mBC, hypertension was the most common comorbidity and highest in Black women. All‐cause mortality risk was lower among those treated with polytherapy vs. monotherapy for hypertension, with the greatest statistically significant attenuation seen among Hispanic patients but also mitigated in Black patients. Our findings point to the importance of awareness and hypertensive management in patients with mBC. To our knowledge, this is one of the first studies to determine whether pharmacologic management of a common comorbidity—hypertension—is associated with mortality in women with mBC.

Although we found that a higher percentage of patients with mBC treated with polytherapy achieved systolic blood pressure control (75.1%) versus monotherapy (24.1%), it is possible that sicker patients with worse mBC may have had lower blood pressure and therefore were more likely to be on monotherapy. It is also possible that clinicians were reluctant to add additional medications to avoid polypharmacy. Additionally, it is feasible that the lower risk of all‐cause mortality in those with polytherapy was due to enhanced monitoring of patients who had hypertension at baseline or developed it after breast cancer diagnosis, even though we adjusted for healthcare utilization during follow‐up. Nonetheless, our study results are consistent with new research demonstrating that women with more metabolic comorbidities have a 44% higher risk of dying from breast cancer and a 53% higher risk of dying from any cause after being diagnosed with early‐stage breast cancer [21]. Taken together, these results point to the importance of monitoring and treating metabolic conditions and hypertension in patients with cancer.

Our findings that all‐cause mortality risk was lower among polytherapy users vs. monotherapy users expands upon those by Chen et al. that examined use of antihypertensive medications and their association with breast cancer recurrence risk [22]. However, that study was limited to a Medicare population with early‐stage breast cancer and lacked information on important confounders such as obesity and dyslipidemia [22]. They found that multiple classes of medications used to treat hypertension did not increase the risk of adverse breast cancer outcomes, including mortality. Their findings are consistent with prior studies examining the influence of antihypertensives on outcomes after diagnosis of early‐stage breast cancer [23, 24, 25, 26]. However, these studies did not evaluate whether the association of antihypertensive use with mortality differs across racial and ethnic groups, nor did they focus on mBC.

Our results also suggest that the reduction in all‐cause mortality risk associated with use of polytherapy for hypertension (vs. monotherapy) was greatest in patients of color, particularly in Hispanic patients and Black patients. Given that hypertension was common among patients of color in this cohort, these findings suggest that such patients may be the most likely to benefit from guideline‐concordant management of hypertension after mBC diagnosis. Breast cancer mortality rates have been declining over time even in patients with newly diagnosed stage IV breast cancer [4, 27]. While the cause of these disparities is certainly multifactorial, our results suggest that comorbidities and their management may play a role. This builds upon our prior work which found that common comorbidities, including hypertension, were associated with an increased mortality risk among women with de novo metastatic disease and may influence racial/ethnic disparities in mortality [7, 13].

Most previously published studies investigating cardiovascular risk factor control have focused on non‐metastatic breast cancer [28], leaving a significant knowledge gap in patients with metastatic disease. Our study points to the importance of awareness of hypertension in this vulnerable population and provides a rationale for a collaboration between oncologists, cardiologists, and primary care teams to develop strategies for detection and management of hypertension. This proactive cross‐specialty approach has been described with monitoring of cancer treatment‐related hypertension [29] and may serve as a helpful model for creating clinical protocols and pathways for blood pressure monitoring and treatment because hypertension might be a harbinger of worse cardiovascular conditions. Finally, our results also open many questions including the choice of antihypertensives and blood pressure goals, as well as the impact of antihypertensive interventions on cancer outcomes that need to be prospectively studied.

Our study had advantages. The cohort had no loss to follow‐up, and all patients were able to be followed to study endpoints (death or study's end). Further, our cohort included nearly 50% patients of color. Importantly, we ascertained medications from pharmacy databases rather than self‐reported data. We also examined a comprehensive set of covariates including other comorbidities, physical activity, tobacco use, BMI, use of other cardiometabolic medications (antilipemics and antidiabetics), healthcare utilization, tumor characteristics, and cancer treatments.

Some potential limitations warrant consideration. Given that this study was based in an integrated healthcare delivery system that measures hypertension management as a part of clinical strategic goals, these results may not be generalizable to other healthcare settings. Nonetheless, the population included in this study reflects the characteristics of cancer survivors living in southern California. This study includes one of the largest cohorts of individuals with mBC from a single community‐based healthcare system and provides important insights into the influence of hypertension management on mortality. Further, this study did not evaluate biologic and lifestyle‐related behaviors (besides smoking history, BMI, and physical activity) that may influence the association of hypertension and other comorbidities with mortality. Finally, this cohort was followed through 2021, and therefore we cannot make inferences about the effectiveness of newer targeted therapies in the metastatic setting. Of note, nearly 28% of the cohort underwent surgery consistent with prior studies of women with mBC [30, 31]. Breast surgeons and medical oncologists might have recommended surgery in certain situations, such as having large tumors causing debilitating pain, in limited metastatic disease sites to prolong remission, or an axillary mass causing nerve and functional damage. Given that all patients were insured, our results may not apply to cancer survivors who are uninsured or under‐insured. Despite these limitations, the study suggests further investigation into whether standard‐of‐care and guideline‐based management for common non‐cancer comorbidities might influence mortality among patients with mBC.

## 5 Conclusion

In conclusion, in this diverse cohort of women with de novo mBC, pre‐existing hypertension was common, ranging from 46.1% in Hispanic patients to 64.6% in Black patients. All‐cause mortality risk was lower among those treated with antihypertensive polytherapy compared with monotherapy, with the greatest statistically significant attenuation in risk seen among Hispanic women as well as a non‐significant decreased risk in Black patients. Effective pharmacologic management of hypertension in women with mBC may help extend life, particularly in patients of color.

## Author Contributions

**Reina Haque:** conceptualization (lead), funding acquisition (lead), investigation (lead), methodology (lead), project administration (lead), resources (lead), supervision (lead), writing – original draft (lead), writing – review and editing (lead). **Amrita Mukherjee:** investigation (supporting), methodology (supporting), writing – review and editing (supporting). **Lie Hong Chen:** data curation (lead), formal analysis (lead), writing – review and editing (supporting). **Tiffany A. Hogan:** conceptualization (supporting), writing – review and editing (supporting). **Moira Brady‐Rogers:** conceptualization (supporting), writing – review and editing (supporting). **Zheng Gu:** data curation (lead), formal analysis (supporting), writing – review and editing (supporting). **Ariel Silverman:** conceptualization (supporting), writing – review and editing (supporting). **Ana Barac:** conceptualization (supporting), investigation (supporting), writing – review and editing (supporting). **Lauren P. Wallner:** conceptualization (equal), funding acquisition (supporting), investigation (equal), methodology (equal), writing – original draft (equal), writing – review and editing (equal).

## Funding

This project was supported by the California Breast Cancer Research Program Grant B28TR5470.

## Disclosure

Preliminary results were presented at the 17th AACR Conference on The Science of Cancer Health Disparities in Racial/Ethnic Minorities and the Medically Underserved, September 2024, Los Angeles, CA.

## Ethics Statement

The study was reviewed by the KPSC Internal Review Board, which waived the right to obtain written or verbal consent from patients for the de‐identified analytic dataset.

## Conflicts of Interest

The authors declare no conflicts of interest.

## Supporting information


**Data S1:** cam471642‐sup‐0001‐DataS1.docx.

## Data Availability

The data that support the findings of this study are available on request from the corresponding author. Data are not publicly available due to privacy or ethical restrictions.

## References

[1] N. A. Howlader and M. Krapcho , “SEER Fast Stats, 1975‐2014,” Stage Distrib. 2018: 2005–14.

[2] C. E. DeSantis , J. Ma , A. Goding Sauer , L. A. Newman , and A. Jemal , “Breast Cancer Statistics, 2017, Racial Disparity in Mortality by State,” CA: A Cancer Journal for Clinicians 67, no. 6 (2017): 439–448, 10.3322/caac.21412. 28972651

[3] C. E. DeSantis , S. A. Fedewa , A. Goding Sauer , J. L. Kramer , R. A. Smith , and A. Jemal , “Breast Cancer Statistics, 2015: Convergence of Incidence Rates Between Black and White Women,” CA: A Cancer Journal for Clinicians 66, no. 1 (2016): 31–42. 26513636 10.3322/caac.21320

[4] J. Ning , S. Peng , N. Ueno , et al., “Has Racial Difference in Cause‐Specific Death Improved in Older Patients With Late‐Stage Breast Cancer?,” Annals of Oncology 26, no. 10 (2015): 2161–2168, 10.1093/annonc/mdv330. 26223248 PMC4576910

[5] D. Huo , H. Hu , S. K. Rhie , et al., “Comparison of Breast Cancer Molecular Features and Survival by African and European Ancestry in the Cancer Genome Atlas,” JAMA Oncology 3, no. 12 (2017): 1654–1662, 10.1001/jamaoncol.2017.0595. 28472234 PMC5671371

[6] R. Yancik , “Cancer Burden in the Aged: An Epidemiologic and Demographic Overview,” Cancer 80, no. 7 (1997): 1273–1283. 9317180

[7] R. Yancik , R. J. Havlik , M. N. Wesley , et al., “Cancer and Comorbidity in Older Patients: A Descriptive Profile,” Annals of Epidemiology 6, no. 5 (1996): 399–412. 8915471 10.1016/s1047-2797(96)00063-4

[8] R. Yancik , “Epidemiology of Cancer in the Elderly. Current Status and Projections for the Future,” Rays 22, no. 1 (1997): 3–9. 9250005

[9] R. P. Hertz , A. N. Unger , J. A. Cornell , and E. Saunders , “Racial Disparities in Hypertension Prevalence, Awareness, and Management,” Archives of Internal Medicine 165 (2005): 2098–2104. 16216999 10.1001/archinte.165.18.2098

[10] D. Braithwaite , D. H. Moore , W. A. Satariano , et al., “Prognostic Impact of Comorbidity Among Long‐Term Breast Cancer Survivors: Results From the LACE Study,” Cancer Epidemiology, Biomarkers & Prevention 21, no. 7 (2012): 1115–1125, 10.1158/1055-9965.EPI-11-1228. PMC3470873 22573797

[11] D. Braithwaite , C. M. Tammemagi , D. H. Moore , et al., “Hypertension Is an Independent Predictor of Survival Disparity Between African‐American and White Breast Cancer Patients,” International Journal of Cancer 124, no. 5 (2009): 1213–1219, 10.1002/ijc.24054. 19058216

[12] D. K. Smith , R. P. Lennon , and P. B. Carlsgaard , “Managing Hypertension Using Combination Therapy,” American Family Physician 101, no. 6 (2020): 341–349. 32163253

[13] L. P. Wallner , L. H. Chen , T. A. Hogan , F. M. Brasfield , and R. Haque , “The Influence of Medical Comorbidities on Survival Disparities in a Multiethnic Group of Patients With De Novo Metastatic Breast Cancer,” Cancer Epidemiology, Biomarkers & Prevention 31, no. 10 (2022): 1935–1943, 10.1158/1055-9965.EPI-22-0065. PMC13721033 35861620

[14] R. Sikka , F. Xia , and R. E. Aubert , “Estimating Medication Persistency Using Administrative Claims Data,” American Journal of Managed Care 11, no. 7 (2005): 449–457. 16044982

[15] H. B. Mehta , S. D. Sura , D. Adhikari , et al., “Adapting the Elixhauser Comorbidity Index for Cancer Patients,” Cancer 124, no. 9 (2018): 2018–2025, 10.1002/cncr.31269. 29390174 PMC5910176

[16] K. Yost , C. Perkins , R. Cohen , C. Morris , and W. Wright , “Socioeconomic Status and Breast Cancer Incidence in California for Different Race/Ethnic Groups,” Cancer Causes & Control 12, no. 8 (2001): 703–711, 10.1023/a:1011240019516. 11562110

[17] R. Tibshirani , “Regression Shrinkage and Selection via the Lasso,” Journal of the Royal Statistical Society. Series B, Statistical Methodology 58, no. 1 (1996): 267–288.

[18] J. K. Vishram‐Nielsen , A. M. D. Kristensen , M. Pareek , et al., “Predictive Importance of Blood Pressure Characteristics With Increasing Age in Healthy Men and Women: The MORGAM Project,” Hypertension 77, no. 4 (2021): 1076–1085, 10.1161/HYPERTENSIONAHA.120.16354. 33641358

[19] NCQA , “Controlling High Blood Pressure,” National Committee for Quality Assurance (NCQA), accessed October 2, 2024, https://www.ncqa.org/hedis/measures/controlling‐high‐blood‐pressure.

[20] B. M. Egan , D. Bandyopadhyay , S. R. Shaftman , C. S. Wagner , Y. Zhao , and K. S. Yu‐Isenberg , “Initial Monotherapy and Combination Therapy and Hypertension Control the First Year,” Hypertension 59, no. 6 (2012): 1124–1131, 10.1161/HYPERTENSIONAHA.112.194167. 22566499 PMC3425944

[21] R. T. Chlebowski , A. K. Aragaki , K. Pan , et al., “Breast Cancer Incidence and Mortality by Metabolic Syndrome and Obesity: The Women's Health Initiative,” Cancer 130, no. 18 (2024): 3147–3156, 10.1002/cncr.35318. 38736319

[22] L. Chen , J. Chubak , D. M. Boudreau , W. E. Barlow , N. S. Weiss , and C. I. Li , “Use of Antihypertensive Medications and Risk of Adverse Breast Cancer Outcomes in a SEER‐Medicare Population,” Cancer Epidemiology, Biomarkers & Prevention 26, no. 11 (2017): 1603–1610, 10.1158/1055-9965.EPI-17-0346. PMC5668179 28807926

[23] P. A. Ganz , L. A. Habel , E. K. Weltzien , B. J. Caan , and S. W. Cole , “Examining the Influence of Beta Blockers and ACE Inhibitors on the Risk for Breast Cancer Recurrence: Results From the LACE Cohort,” Breast Cancer Research and Treatment 129 (2011): 549–556. 21479924 10.1007/s10549-011-1505-3 PMC3145014

[24] G. V. Sørensen , P. A. Ganz , S. W. Cole , et al., “Use of b‐Blockers, Angiotensin‐Converting Enzyme Inhibitors, Angiotensin II Receptor Blockers, and Risk of Breast Cancer Recurrence: A Danish Nationwide Prospective Cohort Study,” Journal of Clinical Oncology 31 (2013): 2265–2272. 23650417 10.1200/JCO.2012.43.9190 PMC3677839

[25] Y. K. Chae , E. N. Brown , X. Lei , et al., “Use of ACE Inhibitors and Angiotensin Receptor Blockers and Primary Breast Cancer Outcomes,” Journal of Cancer 4 (2013): 549–556. 23983819 10.7150/jca.6888 PMC3753529

[26] C. R. Cardwell , U. C. Mc Menamin , B. M. Hicks , C. Hughes , M. M. Cantwell , and L. J. Murray , “Drugs Affecting the Renin‐Angiotensin System and Survival From Cancer: A Population‐Based Study of Breast, Colorectal and Prostate Cancer Patient Cohorts,” BMC Medicine 12 (2014): 28. 24521426 10.1186/1741-7015-12-28 PMC3926686

[27] S. Dawood , K. Broglio , A. M. Gonzalez‐Angulo , A. U. Buzdar , G. N. Hortobagyi , and S. H. Giordano , “Trends in Survival Over the Past Two Decades Among White and Black Patients With Newly Diagnosed Stage IV Breast Cancer,” Journal of Clinical Oncology 26, no. 30 (2008): 4891–4898, 10.1200/JCO.2007.14.1168. 18725649 PMC2736998

[28] A. Howell , A. S. Anderson , R. B. Clarke , et al., “Risk Determination and Prevention of Breast Cancer,” Breast Cancer Research 28, no. 5 (2014): 446, 10.1186/s13058-014-0446-2. PMC4303126 25467785

[29] J. B. Cohen , A. S. Geara , J. J. Hogan , and R. R. Townsend , “Hypertension in Cancer Patients and Survivors: Epidemiology, Diagnosis, and Management,” JACC. CardioOncology 1, no. 2 (2019): 238–251, 10.1016/j.jaccao.2019.11.009. 32206762 PMC7089580

[30] Y. Xie , X. Lv , C. Luo , et al., “Surgery of the Primary Tumor Improves Survival in Women With Stage IV Breast Cancer in Southwest China: A Retrospective Analysis,” Medicine (Baltimore) 96, no. 22 (2017): e7048, 10.1097/MD.0000000000007048. 28562563 PMC5459728

[31] A. C. Bafford , H. J. Burstein , C. R. Barkley , et al., “Breast Surgery in Stage IV Breast Cancer: Impact of Staging and Patient Selection on Overall Survival,” Breast Cancer Research and Treatment 115, no. 1 (2009): 7–12, 10.1007/s10549-008-01. 18581232

