# Supplementary material for: Influence of Hypertension Management on Survival in Patients With Metastatic Breast Cancer
Source: Cancer Med. 2026 Feb 26;15(3):e71642. doi: 10.1002/cam4.71642 (PMC12936983; doi:10.1002/cam4.71642)

| **Supplemental Table 1:** All-cause and breast cancer mortality rates (per 100 person-years) by antihypertensive therapy by race/ethnicity among those with hypertensive medication data (n=704) | | | | | | | | | | |
| --- | --- | --- | --- | --- | --- | --- | --- | --- | --- | --- |
|  | **API** | | **Black** | | **Hispanic** | | **White** | | **Total** | |
|  | **(N=66)** | | **(N=110)** | | **(N=135)** | | **(N=381)** | | **(n=704)** | |
| **All-cause mortality rates per 100 person-years** | |  | |  | |  | |  | |  |
| Monotherapy | 24.3 | | 35.7 | | 27.0 | | 28.1 | | 28.5 | |
| Polytherapy | 22.4 | | 22.9 | | 22.7 | | 20.0 | | 21.4 | |
| Percent difference (%) | 7.6 | | 35.7 | | 15.9 | | 28.7 | | 24.8 | |
|  |  | |  | |  | |  | |  | |
| **Breast cancer mortality rates per 100 person-years** |  | |  | |  | |  | |  | |
| Monotherapy | 24.3 | | 31.1 | | 20.8 | | 24.5 | | 24.8 | |
| Polytherapy | 18.5 | | 19.5 | | 18.9 | | 17.7 | | 18.4 | |
| Percent difference (%) | 23.7 | | 37.3 | | 9.4 | | 27.8 | | 25.8 | |
| ***** Results for women in the Other/Mixed/Unknown group (n=12) are not shown due to small numbers | | | | | | | | | | |

**Supplemental Table 2:** Risk of all-cause and breast cancer mortality in patients with de novo stage IV metastatic breast cancer by antihypertensive medication possession ratio (MPR)

|  | **Antihypertensives**  **MPR ≥ 80%** | **Antihypertensives**  **MPR<80%** |
| --- | --- | --- |
|  | **HR (95% CI)** | **HR (95% CI)** |
| **All-cause mortality** |  |  |
| Crude^a^ |  |  |
| Antihypertensive monotherapy | 1.00 (Ref) | 1.00 (Ref) |
| Antihypertensive polytherapy | 0.46 (0.27-0.80) | 0.62 (0.45-0.84) |
| Adjusted^b^ |  |  |
| Antihypertensive monotherapy | 1.00 (Ref) | 1.00 (Ref) |
| Antihypertensive polytherapy | 0.42 (0.24-0.76) | 0.76 (0.55-1.05) |
| **Breast Cancer mortality** |  |  |
| Crude^a^ |  |  |
| Antihypertensive monotherapy | 1.00 (Ref) | 1.00 (Ref) |
| Antihypertensive polytherapy | 0.45 (0.26-0.78) | 0.62 (0.44-0.86) |
| Adjusted^b^ |  |  |
| Antihypertensive monotherapy | 1.00 (Ref) | 1.00 (Ref) |
| Antihypertensive polytherapy | 0.43 (0.24-0.77) | 0.80 (0.56-1.14) |

^a^Crude model based on time-varying antihypertensive use groups

^b^Multivariable Cox proportional hazard model based on time-varying antihypertensive groups, and adjusted for time-dependent status of antilipemic and antidiabetic use, and baseline age, Neighborhood Deprivation Index, BMI, diabetes, dyslipidemia, Elixhauser Comorbidity Index, insurance payor, annualized outpatient visit, breast cancer subtype, surgery, and palliative cancer treatments (hormonal, chemotherapy, immunotherapy)

**Supplemental Table 3:** Percentage of patients treated with beta-blockers during follow-up by antihypertensive monotherapy and polytherapy status

|  | **Antihypertensive**  **Monotherapy^a^**  **(N, %)** | **Antihypertensive**  **Polytherapy^a^**  **(N, %)** |
| --- | --- | --- |
| **Treated with**  **beta blockers**  Yes  No | 53 (18.8%)  218 (51.7%) | 229 (81.2%)  204 (48.3%) |

^a^ row percentage

**Supplemental Figure 1:** Diagram of primary outcome (all-cause mortality); main exposure variables (antihypertensive drugs); and covariates


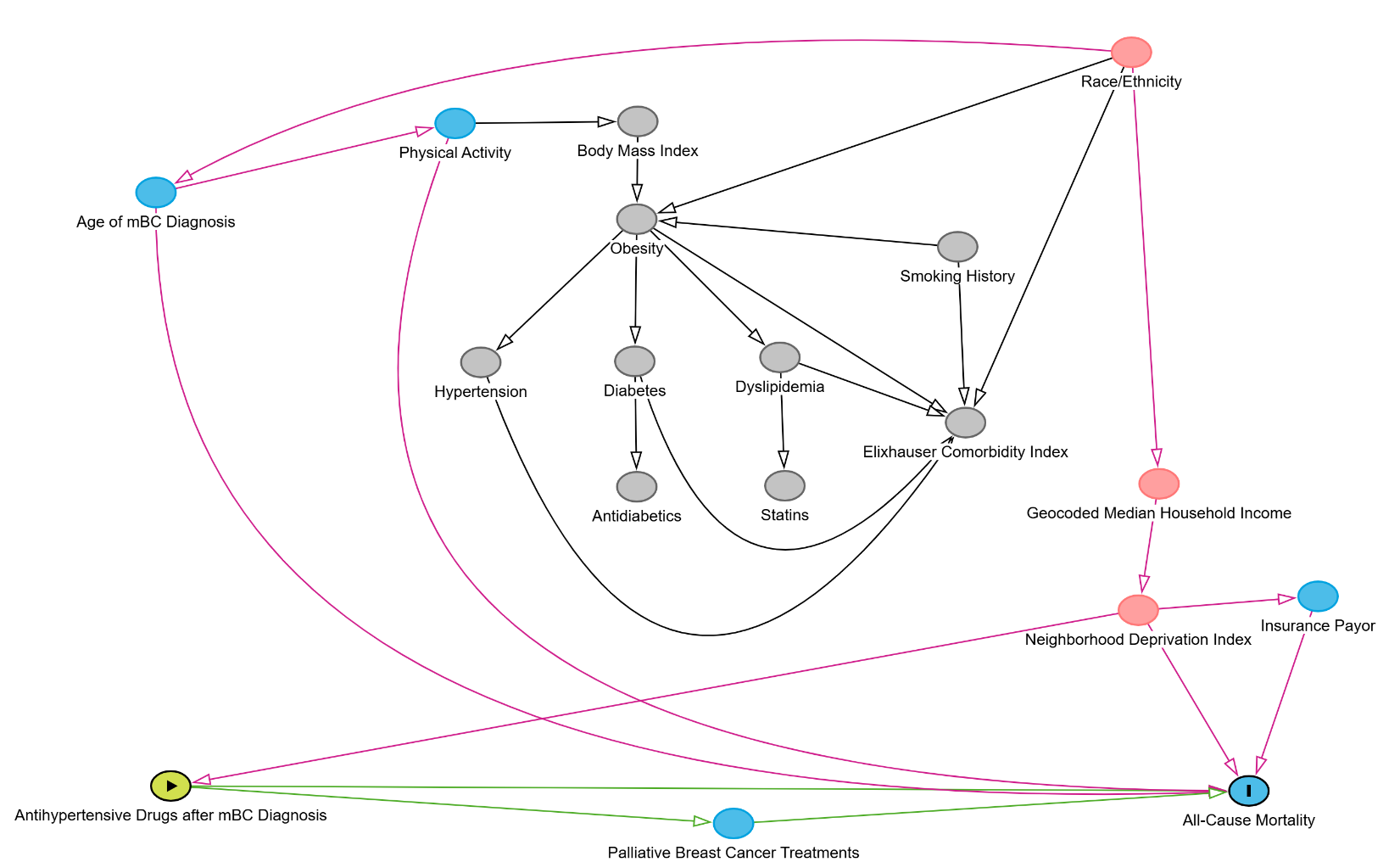

Supplement: Supplementary file 1 — Data S1: cam471642‐sup‐0001‐DataS1.docx. [file CAM4-15-e71642-s001.docx]
